# Standardizing hospital pharmacy practice in home hospitalization: results from a multidisciplinary Delphi consensus in Spain

**DOI:** 10.3389/fpubh.2025.1707726

**Published:** 2025-11-20

**Authors:** Beatriz Somoza-Fernandez, Ana de Lorenzo-Pinto, Vicente Escudero-Vilaplana, Silvia Manrique-Rodriguez, Cristina Villanueva-Bueno, Maria Luisa Martin-Barbero, Carmen Redondo-Galan, Javier Calatayud-Garcia, Victor Gonzalez-Ramallo, Ana Herranz-Alonso, Maria Sanjurjo-Saez

**Affiliations:** 1 Pharmacy Department, Hospital General Universitario Gregorio Marañón, Madrid, Spain; 2 Instituto de Investigación Sanitaria Gregorio Marañón, Madrid, Spain; 3 Home Hospitalization Unit, Hospital General Universitario Gregorio Marañón, Madrid, Spain

**Keywords:** home care services, home hospitalization, hospital pharmacy, pharmaceutical services, patient safety, Delphi technic

## Abstract

**Introduction:**

The expansion of Home Hospitalization (HH) services has prompted the need for standardized pharmaceutical care models to ensure safe and efficient medication management in the home setting. However, Hospital Pharmacy (HP) departments are often heterogeneously involved in HH programs, and there is a lack of consensus on their roles and responsibilities. The objective was to develop a consensus-based activity dashboard to guide HP departments in implementing standardized pharmaceutical care within HH units in Spain.

**Methods:**

A modified Delphi method was conducted in five phases: constitution of a coordinating group, definition of candidate activities, selection of a national expert panel, evaluation of the list of activities (two-round consensus process), and analysis of the results. Experts rated the necessity and feasibility of implementing 44 proposed activities by an HP department, using a 9-point Likert scale (in total, 88 items were rated). Activities were included in the final dashboard if ≥75% of panelists rated both dimensions in the 7–9 range.

**Results:**

A total of 23 multidisciplinary experts participated in the Delphi panel. Consensus was achieved for 60 out of 88 evaluated items (68.2%), with 17 activities rated as both necessary and feasible for inclusion in the final dashboard. These activities spanned six domains: drug dispensing, clinical pharmacy care, risk management, communication and patient education, home administration of antineoplastic agents, and clinical research. Several activities were rated as necessary but lacked feasibility consensus, highlighting systemic and resource-based limitations.

**Conclusion:**

This study provides the first structured consensus on pharmaceutical care activities in HH in Spain, resulting in a practical dashboard to guide HP departments. Its implementation may facilitate the harmonization of care models, may enable the optimization of medication safety, and may support the growing role of pharmacists in home-based care. Addressing feasibility barriers is essential to fully realize the potential of pharmaceutical care in HH programs.

## 1 Introduction

Home hospitalization (HH) units have experienced an extraordinary period of growth over the past few years. Increasing evidence and advancements in digital health technologies have positioned the HH model as a promising strategy to reduce healthcare spending and to improve patient outcomes (1).

HH aims to provide patients and families with an alternative to conventional hospitalization for the delivery of care and treatment. By releasing physical beds, HH contributes to building greater capacity for inpatient hospitalization (2). Patients receiving HH have shown greater mobility, lower incidence of delirium, better anxiety and depression scores, and a reduction in the incidence of healthcare-associated infection (3–5). In addition, patient and family satisfaction increases due to their greater involvement in patient care and the humanized approach to care delivery (6).

Globally, HH services are diverse in scope and implementation. As a result, HH pharmacotherapeutic services are delivered in a heterogeneous case-mix of healthcare scenarios, defined both by local hospital practices, pharmacy-related regulations, and differing definitions of HH. In Spain, HH programs typically operate under hospital-level regulation and are conceived as an acute care service equivalent to inpatient hospitalization, both operationally and in terms of reimbursement.

Hospitals worldwide have adopted diverse homecare models—with some focused exclusively on oncological, post-surgical, or orthopedic care and others focused on targeting chronic disease management or home-based rehabilitation (7–9). Given this global variability, the level of involvement of Hospital Pharmacy (HP) departments in HH also varies considerably. It has been reported that only 65.9% of hospitals offering HH services are supported by an actively involved HP department (10). Although this involvement is far from implying widespread participation, the support of HP departments is considered an essential factor for the success of home care (11, 12). Integrating a pharmacist into an HH unit has been shown to have a positive impact on patients’ safety and to reduce medication costs by enabling the detection of medication discrepancies, medication deprescribing, and the management of home parenteral therapy (13). However, few specific programs have been published worldwide describing how to integrate a pharmacist holistically into a non-monographic HH unit.

These controversies and the subsequent lack of consensus may preclude standardization and continuous quality improvement of HH pharmacotherapy. Even the “Home Hospital Pharmacy Playbook” by the American Society of Health-System Pharmacists (ASHP) highlights the need for global collaboration among pharmacists and concurrently drives the legislation that supports the appropriate dispensing models in home healthcare (11). However, the ASHP guide refers broadly to pharmacy services in home care, encompassing a variety of models that may differ significantly from the HH model, as defined within the Spanish healthcare system. In this context, the rapid expansion of HH services is currently pushing HP departments to define and prioritize their roles in alignment with the demands of real-world, acute-level care delivered at home. In this context, given the absence of a globally accepted pharmaceutical care model in HH, developing a national consensus on essential pharmacy activities can provide a foundation for standardizing care and improving quality within specific health systems.

To define a gold standard pharmaceutical care model for patients receiving hospital-at-home services, it is essential to identify the key actions to be undertaken by HP departments and to clearly delineate the roles and responsibilities of pharmacists, nurses, and clinicians in medication management. The current study aimed to develop a consensus-based activity dashboard to guide HP departments in the implementation of a standardized pharmaceutical care program for HH patients within the Spanish healthcare context.

## 2 Materials and methods

The study was carried out in five phases following the modified Delphi methodology: constitution of the coordinating group, definition of candidate activities, selection of a Spanish national expert panel, evaluation of the list of activities (two-round consensus process), and analysis of the results to define the final activity dashboard.

### 2.1 Constitution of the coordinating group

The coordinating group comprised nine healthcare professionals belonging to a tertiary hospital. All of them were selected for their established and long-standing experience in the management of HH pharmacotherapy. For this study, “experience” was defined as at least 3 years of active involvement in HH services and/or having participated in the implementation of new services within the HH unit, ensuring both practical expertise in patient care and knowledge of organizational processes.

Seven members were hospital pharmacists, two with specific expertise in the home care of onco-hematological patients, two in pediatrics, two in complex chronic patients, and one in psychiatric care. To ensure a multidisciplinary perspective, the group also included one HH physician and one HH nurse.

The coordinating group was tasked with drafting the items to be included in the study’s questionnaire, interpreting the results, and critically reviewing the final report.

### 2.2 Definition of candidate activities

The coordinating group conducted a comprehensive literature research and review that focused on reported HP activities developed in HH units. Searches of MEDLINE and EMBASE databases from inception to July 2024 were performed using the keywords: [“home hospitalization” OR “home-care” OR “hospital at home” OR “home based care”] AND [“pharmacy” OR “clinical pharmacy” OR “hospital pharmacy”]. Seven publications were identified from which information was extracted (12–18). In addition, the ASHP guide “Home Hospital Pharmacy Playbook” (11) and Joint Commission International Standards for Home Care (19) were reviewed. Based on the results of this literature review and the coordinating group experience, a list of 44 potential activities to be developed by HP departments was identified. Six theme-based blocks were set out: (1) dispensing; (2) clinical pharmacy care; (3) risk management; (4) effective communication among HH staff and patient information; (5) home administration of antineoplastic agents; and (6) clinical research.

### 2.3 Selection of a national expert panel

We identified Spanish hospitals with an HH unit and selected those with ≥600 beds to represent mature programs. Larger institutions were prioritized because they generally provide a broader range of services and more complex, structured models of care. In total, 30 hospitals met this criterion. From each center, the hospital pharmacist responsible for the HH unit was invited to participate.

To reach a multidisciplinary perspective on the panel, the coordinating group invited three internal medicine physicians and two nurses to participate in the Delphi model. They were identified as national opinion leaders and contributors to key publications in the field.

Participants were presented with the information about the study via email, and informed consent was assumed if the participant commenced the survey. The surveys were also sent via email.

The survey began with a brief questionnaire concerning the primary characteristics of the hospitals and HH units to which the panelists belonged to: hospital location, hospital bed size, number of HH inpatients (patients receiving acute-level care at home) per day, activities performed by HH units, and types of patients admitted in HH units. Variables related to domiciliary medication management were also collected: drugs being provided by the HP department during hospitalization (acute and/or chronic medications), medications dispensing models (collective and/or individualized dispensing systems), and basic activities developed by HP departments.

Participants’ years of professional involvement in HH were also collected. A minimum of 3 years of experience in the field was required for inclusion criterion; participants with less experience were not eligible for inclusion in the panel.

### 2.4 Evaluation of the list of scenarios

A two-round Delphi survey was conducted between July and November 2024. A panel of participants scored the “Necessity” and “Feasibility” of implementing each activity on a 9-point Likert scale, following common Delphi and RAND/UCLA Appropriateness Method practices. In this way, experts analyzed 88 items (both “N” and “F” for a total of 44 activities). The level of agreement was classified as 1–3 (disagree), 4–6 (neither agree nor disagree), or 7–9 (agree). Hence, the higher the score, the higher the level of agreement concerning “N” and “F” of implementing each activity.

Definitions of “N” and “F” were provided to the panelists to standardize their understanding during scoring. “N” was defined as the importance of implementing the activity to ensure optimal pharmaceutical care in HH, while “F” referred to the practicality of implementing the activity. Feasibility was evaluated considering current legal, structural, and operational conditions within the Spanish public healthcare system, including existing reimbursement schemes and regulatory allowances for hospital pharmacists participating in HH care.

Consensus was reached for each response when ≥75% of the participants rated the activity in the upper or lower third of the scale (1–3, no necessary and/or no feasible, and 7–9, necessary and/or feasible). In total, the activities receiving a median score of “N” and/or “F” falling within the Likert scale categories of 1–3 were designated for “exclusion.” The activities receiving a median score of 7–9 (both “N” and “F”) were designated for “inclusion.” Items not meeting these criteria were deemed “borderline.”

In addition to rating each candidate activity, the panelists could provide qualitative feedback on the title and description of each candidate activity. After the first round, the coordinating group revised the wording of any items that required improvement or clarification.

In round 2, participants were asked to review the items that had not reached a consensus for exclusion or inclusion (“borderline items”) and to rate them again. The median and range values for each activity were provided to participants, so they could reconsider their answers.

### 2.5 Analysis of the results

Data were analyzed anonymously using Stata version 18 for the analysis of median and range values around each candidate outcome, based on all participating respondents. Missing answers were regarded as non-participation. Demographic characteristics were analyzed using descriptive statistics.

## 3 Results

### 3.1 Panelists

In total, 35 national experts were invited to complete the questionnaire (30 hospital pharmacists, 3 internal medicine physicians, and 2 nurses). Twenty-five experts (71.4%) accepted the invitation. Of them, 23 (92%) completed both Delphi rounds (19 hospital pharmacists, 3 internal medicine physicians, and 1 nurse).

All of them possessed a minimum of 3 years of professional experience in the field of HH (median: 10 years; range: 5–30 years).

Experts belonged to 19 different hospitals; 5 of them (26%) were located in Catalonia, 4 (21%) in Madrid, 3 (16%) in Galicia, 2 (11%) in the Basque Country, and 1 each in Andalusia, Balearic Islands, Canary Islands, Cantabria, and Valencian Community. The median hospital bed size was 849 (range 680 to 1,500).

### 3.2 Home hospitalization units and hospital pharmacy departments

Regarding the HH units included, the median number of inpatients per day was 55 (range 20 to 112). All of them included, among the activities performed, the administration of parenteral antimicrobial agents and the management of chronic disease exacerbations. Wound care and palliative care were provided by 11 (57.9%) and 6 (31.6%) HH units, respectively. Sixteen units (84.2%) included oncohematological inpatients, whereas pediatric and psychiatric inpatients were admitted by 9 (47.4%) and 7 (36.8%) HH units, respectively. Additionally, 9 (47.4%) HH units administered parenteral antineoplastic agents at home.

Key issues concerning pharmacotherapy management were the following: 11 (58%) HP departments only dispensed acute medications prescribed during hospitalization, whereas 4 (21.0%) units provided the whole treatment, including patients’ chronic medications. Four (21.0%) HP departments referred to work with a different dispensing model.

Five (26%) HP departments exclusively used a collective dispensing system (medications are distributed based on nurse requests), and 14 (74%) included mixed models combining collective and individualized dispensing systems.

Medication reconciliation at admission was conducted by a hospital pharmacist in 7 (36.8%) HH units. Daily review of medical prescriptions was performed by 16 (84.2%) HP departments, 9 (56.2%) of them only analyzed acute medications prescribed during hospitalization, and 7 (43.8%) of them reviewed the whole treatment, including patients’ chronic medications.

Intravenous medication compounding was developed by 17 (89.4%) HP units.

### 3.3 Delphi results

In the first round, consensus was reached for 54 out of the 88 items analyzed (61.4%). After the two Delphi rounds, consensus was reached for 60 items (68.2%), while there was a discrepancy in 28 items (31.8%).

Seventeen activities out of the 44 proposed ones (38.6%) reached necessity and feasibility consensus and were included in the final dashboard (Table 1). To support the practical adoption of the final dashboard, the 17 validated pharmaceutical care activities to be implemented by HP were grouped into four implementation-oriented categories (Table 2).

**Table 1:** Final results from the Delphi study.

Activities to include in the final dashboard	Necessity			Feasibility			Final result
	Likert score; median (range)	Participants ranging the item with 7–9 (% (*n*))	Participants ranging the item with 1–3 (% (*n*))	Likert score; median (range)	Participants ranging the item with 7–9 (% (*n*))	Participants ranging the item with 1–3 (% (*n*))	
1. Drug selection and dispensing activities							
**1.1. Drug evaluation and inclusion into the hospital’s Pharmacotherapeutic Guide**	9 (3–9)	90.5% (19)	9.5% (2)	8 (3–9)	90.5% (19)	4.8% (1)	**N and F consensus**
**1.2. Update of the list of medications stocked and used in the home care organization**	9 (2–9)	95.2% (20)	4.8% (1)	9 (6–9)	95.2% (20)	0 (0)	**N and F consensus**
**1.3. Acute medications dispensing**	8 (6–9)	85.7% (18)	9.5% (2)	9 (1–9)	90.5% (19)	9.5% (2)	**N and F consensus**
1.4. Whole treatment dispensing (including chronic medications)	5 (1–9)	38.1% (8)	38.1% (8)	4 (1–9)	38.1% (8)	42.9% (9)	N and F discrepancy
1.5. Medication dispensing by a collective system (based on nurses’ requests)	7 (1–9)	61.9% (13)	19.0% (4)	8 (1–9)	81.0% (19)	4.8% (1)	N discrepancy F consensus
1.6. Medication dispensing by personalized dispensing systems (“pill box”)	2 (1–9)	14.3% (3)	57% (12)	2 (1–9)	4.8% (1)	86% (18)	N discrepancy no-F consensus
1.7. Medication dispensing by automated dispensing systems located in the unit	9 (7–9)	95.2% (20)	5% (1)	6 (2–9)	47.6% (10)	14% (3)	N consensus F discrepancy
1.8. Therapeutic substitution of chronic medications for those available at the hospital	4 (1–8)	23.8% (5)	43% (9)	7 (1–8)	52.4% (11)	24% (5)	N and F discrepancy
2. Clinical Pharmacy care							
2.1. Medication reconciliation at admission	9 (4–9)	95.2% (20)	0 (0)	7 (2–9)	61.9% (13)	19% (4)	N consensus F discrepancy
**2.2. Pharmaceutical recommendations for the acute treatment optimization**	9 (6–9)	95.2% (20)	0 (0)	8 (3–9)	76.2% (16)	4.8% (1)	**N and F consensus**
2.3 Pharmaceutical recommendations for the whole chronic treatment optimization	8 (2–9)	81.0% (17)	9.5% (2)	6 (1–9)	33.3% (7)	19% (4)	N consensus F discrepancy
**2.4. Daily review of acute medications prescribed during hospitalization**	8 (2–9)	81.0% (17)	19% (4)	8 (1–9)	76.2% (16)	14.3% (3)	**N and F consensus**
2.5. Daily review of the whole treatment (including new chronic medications prescribed during hospitalization)	8 (2–9)	81.0% (17)	14.3% (3)	6 (2–9)	47.6% (10)	28.6% (6)	N consensus F discrepancy
2.6. Pharmacokinetics and therapeutic drug monitoring	8 (5–9)	95.2% (20)	0 (0)	7 (2–9)	61.9% (13)	19% (4)	N consensus F discrepancy
**2.7. Implementation of antimicrobial stewardship programs**	9 (6–9)	95.2% (20)	0 (0)	8 (4–9)	81.0% (17)	0 (0)	**N and F consensus**
2.8. Medication reconciliation at discharge	8 (4–9)	90.5% (19)	0 (0)	6 (1–9)	38.1% (8)	38.1% (8)	N consensus F discrepancy
2.9. Pharmacotherapeutic information to patients and caregivers about their treatments at discharge	8 (3–9)	85.7% (18)	4.8% (1)	5 (2–8)	23.8% (5)	42.9% (9)	N consensus F discrepancy
2.10. Medication compliance monitoring after discharge	7 (1–9)	57.1% (12)	14.3% (3)	5 (2–8)	0 (0)	90.5% (19)	N discrepancy no-F consensus
2.11. Remote patient monitoring: implementation of an e-healthcare tool (actions provided by the e-healthcare tool or remote monitoring activities are described below)							
Medication compliance monitoring using an e-healthcare tool	7 (2–9)	76.2% (16)	9.5% (2)	5 (1–8)	19.0% (4)	47.6% (10)	N consensus F discrepancy
Medication intake notifications by the e-health tool	7 (2–9)	81.0% (17)	4.8% (1)	5 (1–8)	14.3% (3)	47.6% (10)	N consensus F discrepancy
Self-recording for vital signs in the e-health tool	7 (1–9)	61.9% (13)	19% (4)	5 (2–9)	28.6% (6)	33.3% (7)	No-N and no-F consensus
Remote patient monitoring by wearable health devices	7 (2–9)	76.2% (16)	4.8% (1)	5 (1–8)	38.1% (8)	33.3% (7)	N consensus F discrepancy
Continuous two-way communication (pharmacist-patients/caregivers) for solving pharmacotherapeutic questions	8 (2–9)	81.0% (17)	4.8% (1)	5 (2–8)	23.8% (5)	42.9% (9)	N consensus F discrepancy
Medication-related problems and adverse events registration by patients/caregivers	5 (2–8)	23.8% (5)	42.9% (9)	6 (1–8)	38.1% (8)	14.3% (3)	N and F discrepancy
3. Risk management plan							
**3.1. Adverse events registration**	8 (7–9)	100% (21)	0 (0)	8 (3–9)	76.2% (16)	14.3% (3)	**N and F consensus**
3.2. Medication storage areas in patients’ homes evaluation to ensure that medications are stored properly	7 (1–9)	76.2% (16)	4.8% (1)	2 (1–4)	0 (0)	90.5% (19)	N consensus No-F consensus
**3.3. Clinical practice meetings and committees engagement**	9 (5–9)	95.2% (20)	0 (0)	8 (4–9)	85.7% (18)	0 (0)	**N and F consensus**
3.4. Effective communication with community healthcare professionals for ensuring continuity of care	8 (5–9)	95.2% (20)	0 (0)	6 (2–9)	38.1% (8)	28.6% (6)	N consensus F discrepancy
**3.5. Centralized preparation of parenteral hazardous drugs**	9 (8–9)	100% (21)	0 (0)	9 (4–9)	95.2% (20)	0 (0)	**N and F consensus**
3.6. Centralized preparation of parenteral non-hazardous drugs	9 (5–9)	85.7% (18)	0 (0)	7 (1–9)	52.4% (11)	19.0% (4)	N consensus F discrepancy
4. Effective communication among HH staff and patient information							
**4.1. Staff training for the safe medication administration**	9 (7–9)	100% (21)	0 (0)	8 (5–9)	90.5% (19)	0 (0)	**N and F consensus**
4.2. Patients/caregivers’ education about how to properly and safely store medications at home	8 (5–9)	90.5% (19)	0 (0)	6 (1–9)	19.0% (4)	33.3% (7)	N consensus F discrepancy
4.3. Patients/caregivers education about how to properly and safely store and administer hazardous medications	9 (5–9)	95.2% (20)	0 (0)	6 (2–9)	38.1% (8)	23.8% (5)	N consensus F discrepancy
4.4. Patients/caregivers education about how to properly and safely store and administer high-risk medications	9 (5–9)	95.2% (20)	0 (0)	6 (2–9)	38.1% (8)	23.8% (5)	N consensus F discrepancy
**4.5. Implementation of self-administration of intravenous drug programs**	9 (7–9)	100% (21)	0 (0)	8 (4–9)	85.7% (18)	4.8% (1)	**N and F consensus**
**4.6. Staff and patients training for reporting suspected adverse events**	8 (5–9)	90.5% (19)	0 (0)	8 (5–9)	90.5% (19)	0 (0)	**N and F consensus**
5. Antineoplastic agents administration at home							
**5.1. Active involvement in the program coordination group**	9 (1–9)	95.2% (20)	4.8% (1)	8 (2–9)	76.2% (16)	4.8% (1)	**N and F consensus**
**5.2. Selection of chemotherapy drugs that can potentially be suitable for home administration**	9 (4–9)	95.2% (20)	0 (0)	8 (2–9)	81.0% (17)	4.8% (1)	**N and F consensus**
**5.3. Development of a comprehensive standardized protocol of the process, including centralized preparation, drug transportation, home administration, and drug waste management**	9 (8–9)	100% (21)	0 (0)	8 (6–9)	95.2% (20)	0 (0)	**N and F consensus**
**5.4. Development of specific protocols for the home administration of every drug**	9 (6–9)	95.2% (20)	0 (0)	8 (5–9)	81.0% (17)	0 (0)	**N and F consensus**
**5.5. Staff training for the safe medication administration and management of hazardous drugs at home**	9 (7–9)	100% (21)	0 (0)	8 (3–9)	81.0% (17)	4.8% (1)	**N and F consensus**
5.6. Patients/caregivers training for the early detection and management of adverse events	9 (5–9)	95.2% (20)	0 (0)	7 (1–9)	52.4% (11)	19.0% (4)	N consensus F discrepancy
5.7. Detection of treatment toxicity through remote monitoring	8 (5–9)	81.0% (17)	0 (0)	6 (1–9)	38.1% (8)	23.8% (5)	N consensus F discrepancy
6. Clinical researching							
6.1. Drug stability testing in order to provide novel data for their administration at home	9 (5–9)	95.2% (20)	0 (0)	5 (1–9)	28.6% (6)	28.6% (6)	N consensus F discrepancy

Activities included in the final dashboard are indicated in bold. N/F consensus: necessary/feasible activity by consensus; no-N/no-F consensus: no-necessary/no-feasible activity by consensus; N/F discrepancy: consensus was not reached.

**Table 2:** Final validated pharmaceutical care activities for home hospitalization grouped by implementation categories.

Foundational/operational activities	Drug evaluation and inclusion into the hospital’s Pharmacotherapeutic Guide Update of the list of medications stocked and used in the home care organization Acute medication dispensing Centralized preparation of parenteral hazardous drugs
Core clinical pharmacy services	Pharmaceutical recommendations for the acute treatment optimization Daily review of acute medications prescribed during hospitalization Implementation of antimicrobial stewardship programs
High-risk medication management	Adverse events registration Staff and patients are training for reporting suspected adverse events
Coordination, training and governance activities	Staff training for safe medication administration Implementation of self-administration of intravenous drug programs Clinical practice meetings and committees engagement With respect to the program of antineoplastic agents administration at home: Active involvement in the program coordination group Selection of chemotherapy drugs that can potentially be suitable for home administration Development of a comprehensive standardized protocol of the process, including centralized preparation, drug transportation, home administration and drug waste management Development of specific protocols about the home administration of every drug Staff training for the safe medication administration and management of hazardous drugs at home

## 4 Discussion

To the best of our knowledge, this is the first expert consensus concerning HH pharmacotherapy management and HP activities in Spain. The Delphi technique is a widely recognized and robust process that has been well-established as a valid method to build consensus around clinical and practical issues. This Delphi consensus provides a multidisciplinary and real-life perspective on medication management at home.

### 4.1 Drug selection and dispensing activities

The gold standard model that emerged from this consensus includes similar essential activities that HP already develops for conventional hospitalization, concerning drug evaluation and overall management of stocks. However, some differences were found regarding which medications should be dispensed by the hospital.

The experts did not reach a consensus about dispensing usual chronic medications to HH patients. In conventional inpatient care, medications are typically supplied by the HP department; consequently, the use of patient-owned medications is minimized for safety reasons. In HH programs, if the HP department does not provide chronic medications, responsibility may fall to patients themselves. Formal processes are often unclear regarding how to safely manage the concurrent use of patient-owned and hospital-supplied medications at home.

Although the Joint Commission International Standards for Home Care are not specific to HH programs, their principles regarding safe medication management are relevant and applicable to HH settings, which also involve delivering healthcare services at patients’ homes. These standards do not specify who should supply these medications, but they emphasize the need for HH units to implement procedures to ensure safe medication use and storage in the home (19). Practical strategies to achieve these safety goals have been described; for example, Niehoff et al. reported a home-care pharmacy program in which outpatient medications were stored in a designated, consistent location, separate from any drug provided by medical staff, thereby reducing the risk of duplication or confusion (15).

Concerning medications provided by the hospital, it has been shown that automated dispensing devices improve dispensing efficiency and significantly reduce the rate of medication errors related to the dispensing process for conventional hospitalization units (20). However, no previous studies were published for HH care, and Joint Commission International Standards do not include any recommendations about this item (19). In Niehoff et al. HH program (15), all medications provided by the hospital were packaged in the pharmacy in unit doses for single use with specific instructions about the administration. On the other hand, Webster et al. described the implementation of a pharmacy program in their HH unit using automated dispensing devices (16).

### 4.2 Clinical pharmacy care

There is a high percentage of clinical activities rated as necessary that did not reach a consensus concerning their feasibility to be implemented. Some of these activities are widely considered to be gold standard practices in conventional hospitalization, such as medication reconciliation at admission and discharge, daily review of prescriptions, or therapeutic drug monitoring (21, 22). These activities have also been shown to have a positive impact on ensuring patient safety and pharmacotherapy optimization in HH units. Belaiche et al. conducted a study to assess the rates of drug-related problems (DRPs) prevented by a pharmacist in a home-based hospital unit in British Columbia (17); they found a total of 9.4% of patients with a DRP (n = 2,878). The pharmacist’s recommendations were accepted in 87.6% of cases. Brito et al. reported an acceptance rate of 96.3% for the pharmacist recommendations (18). Emonds et al. conducted a prospective study that enrolled all patients admitted to their HH unit for 1 year (n = 102) (13). They found that the cost avoided by identifying and deprescribing inappropriate medications by a hospital pharmacist was approximately $51,000.

Some activities related to the remote monitoring of our patients’ pharmacotherapy by an e-healthcare tool were proposed to experts. Most of them were considered necessary to be implemented, but no consensus was reached concerning their feasibility. Initial costs incurred for its implementation may arise as a primary cause of it. There is currently no published evidence assessing the cost-effectiveness of remote monitoring programs for HH patients. However, telepharmacy has been shown to improve treatment adherence and patient use of medicines, reduce adverse events and resource consumption, and enhance health-related quality of life in patients with chronic conditions (23). This tool may be particularly helpful for the pharmacotherapeutic management of patients with chronic diseases that require acute care at home. For example, Niehoff et al. provided, as part of their program, video telehealth consultations during the entirety of the inpatient stay, using a hospital-provided tablet device (15). Webster et al. performed virtual medication inventory visits for each admission to ensure the home medication list was accurate (16).

### 4.3 Risk management plan

Certain distinctive characteristics of the home healthcare environment may affect patient safety. However, on account of formulating a comprehensive risk management plan, HH has been associated with improved patient safety outcomes compared to conventional hospital care (24, 25). The involvement of HP in clinical practice meetings and committees was identified by this Delphi model as a basic activity to ensure patients’ safety.

Risk management plans must also involve a specific procedure concerning sterile formulation safety, including parenteral drugs. The current increasing complexity and variability of the compounding activity in hospitals could lead to potential drug administration errors, and centralized compounding of parenteral drugs in HP departments has been shown to reduce the incidence of medication errors and improve cost savings (26). In this context, centralized preparation of parenteral hazardous drugs by the HP department was considered a necessary and feasible activity. On the other hand, centralized preparation of parenteral non-hazardous drugs was rated a necessary task, but there was no consensus about its feasibility. However, Emonds et al.’s study found that, for 102 patients enrolled during 1 year, the pharmacist managed 104 days of home IV therapy, resulting in cost savings of approximately USD$17,000 (13). They coordinated intravenous preparation and medication delivery with HH nurses and pharmacy personnel and created policies and procedures for IV medication preparation, dispensing, and transport in line with local facility and admixture stability standards.

### 4.4 Effective communication among home hospitalization staff and patient information

Effective communication and coordination between healthcare professionals and patients or caregivers is of the essence for ensuring HH safety. Specific staff training for the safe administration of drugs at home was rated as a necessary and feasible activity to be implemented by the HP department. Joint Commission International Standards for Home Care state that staff involved in medication use must be granted access to appropriate sources of drug information, including preparation and compounding of sterile products, medication dispensing, and administration (19).

Patient education about medication management and administration did not reach a feasibility consensus among our experts. However, Joint Commission International standards include patient/caregiver education about medication storage, preparation, and administration as a basic standard (19). Emonds et al. (13) scheduled video or telephone visits by a pharmacist for education about medication use, such as device training for glucometers or inhalers, and use of pillboxes. If pharmacists in an HH unit find patient education as an unfeasible goal to be developed, a multidisciplinary distribution of tasks and collaborative working seems to set the gold standard for such a crucial item. In addition, according to Joint Commission International Standards, the staff should draw specific protocols about the correct use of controlled substances, emergency medications, or investigational products at home and implement educational strategies concerning these items (19).

### 4.5 Administration of antineoplastic agents at home

Home administration programs of oncologic therapies must comply with the same quality standards as hospital administration (27, 28). However, pharmaceutical and clinical characteristics of the administered treatments at home, including their posology, stability, route and duration of administration, and safety profile, become particularly important to consider. Procedures for safe handling of medications at home, such as disposal of waste, safe management of unused medications, or clean-up of drug spills, need to be explicitly defined, as described by Joint Commission International standards (19).

Oncology, HH, and HP departments must be coordinated for drug prescription, validation, drug administration, detection of adverse events, and patient follow-up. This way, experts agree that HP departments should take an active part in project implementation.

Patients and caregivers should receive educational and supporting material about the home-based program, but this task did not reach a feasibility consensus among our Delphi experts. Patient information concerning the correct management of hazardous drugs at home demands a multidisciplinary approach to develop a holistic pharmacotherapeutic care plan for the whole drug management process.

### 4.6 Clinical research

Outpatient Parenteral Antimicrobial Therapy (OPAT) programs have been implemented as a useful healthcare tool worldwide that enables patients to receive optimal antimicrobial treatments at home (29, 30). One of the most challenging tasks of these programs is translating hospital-routine antimicrobial regimens to the outpatient setting. The lack of antimicrobial stability data has significant implications for the selection and stewardship of antimicrobial agents suitable for home administration.

Scientific communities worldwide demand further stability studies to ensure optimal patient outcomes and to increase the number of patients who could be treated in an OPAT regimen. Drug stability testing to provide novel data for their administration at home was rated by our Delphi experts as a necessary task, but they did not reach a consensus concerning the feasibility of implementing this measure.

In this context, it is known that the increasing demand for care, lack of time to implement new ideas, and inadequate facilities are perceived as the most significant barriers for performing clinical research activities at hospitals (31).

### 4.7 Relevant activities not included in the final dashboard

This dashboard for pharmaceutical care at home could help optimize therapeutic medication management and health outcomes in HH patients. Our study found strong consensus for 17 core activities to be implemented by HP departments as the basis for planning a pharmaceutical care procedure for any HH unit in Spain.

However, a total of 20 activities (45.5%) were considered necessary to be implemented, but experts rated them as unfeasible, or consensus about their feasibility was not reached. Panelists’ results suggest that workload and health-system funding preclude the feasibility of implementing some crucial activities, particularly in the context of competing priorities for limited pharmacy resources, both material and human. Some practices widely recognized as beneficial were excluded due to these feasibility concerns. Notable examples include medication reconciliation and therapeutic drug monitoring (TDM), both of which are well-established patient safety interventions in conventional hospitalization. Their exclusion from the final dashboard does not reflect a lack of clinical value, but rather highlights real-world operational barriers in the HH setting.

This finding reveals a gap between best practices and current implementation capacity. Recognizing the importance of these excluded activities ensures that the dashboard remains a flexible and evolving tool that is adaptable to local improvements, local infrastructure, and coordination.

### 4.8 The role of HP departments

Hospital pharmacists should be provided a prominent role in creating optimal medication management protocols at home, by describing efficient dispensing models, by establishing drug administration rules for health staff and patients/caregivers, or by providing enhanced digital tools for drug monitoring. The contributions of HP departments are invaluable to patient care in the hospital setting, unique from but complementary to those of other health disciplines (16). It is undeniable that pharmacy practice is witnessing a transformative era, and HP departments are increasingly embracing a new role specifically for home hospital patients that needs to be promoted.

The main limitation is that the dashboard reflects consensus specific to the Spanish healthcare context; consequently, its feasibility in other systems may be affected by differences in reimbursement policies, licensure requirements, and regulatory frameworks. Nevertheless, the dashboard’s methodology and structure provide a transferable foundation for adaptation in other countries, provided they are aligned with local regulatory and operational conditions and, where necessary, with the prevailing definition of HH. Second, as a consensus-based tool, the dashboard shows experts’ points of view, and some aspects, such as the resources available, may condition the inclusion of activities in the dashboard. Finally, we restricted inclusion to hospitals with ≥600 beds to preferentially recruit mature, consolidated HH units that deliver a broad range of pharmaceutical-care activities. However, this selection criterion may limit the generalizability of the dashboard to smaller or monographic HH units, which are also present in the healthcare system. In such cases, local adaptation and prioritization of activities may be required based on the specific resources, organizational structure, and patient population of each institution.

## 5 Conclusion

This Delphi consensus study has led to the development of a structured activity dashboard aimed at guiding Hospital Pharmacy (HP) departments in Spain in the implementation of standardized pharmaceutical care in Home Hospitalization (HH) units. A total of 17 activities were identified as both necessary and feasible by a multidisciplinary panel of experts and should serve as the foundation for a harmonized pharmacotherapeutic model in this care setting.

The proposed dashboard reflects a real-world consensus on key domains such as drug selection and dispensing, clinical pharmacy services, risk management, communication, and home administration of antineoplastic agents. Notably, several additional activities were deemed essential yet lacked feasibility consensus, highlighting existing structural and resource limitations across healthcare institutions.

Future strategies should prioritize the integration of hospital pharmacists into HH teams and support the adoption of digital tools, remote monitoring systems, and research capabilities to overcome implementation barriers. Standardizing pharmaceutical care in HH has the potential to improve medication safety, optimize therapeutic outcomes, and enhance the overall quality of care delivered to patients in the home setting.

Although this framework was developed within the Spanish healthcare system, it may serve as a reference point for other health systems aiming to structure pharmaceutical care in similar models. However, any application outside of Spain must be carefully contextualized, considering local regulatory environments, reimbursement mechanisms, and healthcare delivery structures.

## Data Availability

The raw data supporting the conclusions of this article will be made available by the authors, without undue reservation.
